# Excitatory Repetitive Transcranial Magnetic Stimulation Over the Ipsilesional Hemisphere for Upper Limb Motor Function After Stroke: A Systematic Review and Meta-Analysis

**DOI:** 10.3389/fneur.2022.918597

**Published:** 2022-06-20

**Authors:** Zhiqing Tang, Kaiyue Han, Rongrong Wang, Yue Zhang, Hao Zhang

**Affiliations:** ^1^School of Rehabilitation, Capital Medical University, Beijing, China; ^2^Beijing Bo'ai Hospital, China Rehabilitation Research Center, Beijing, China; ^3^Cheeloo College of Medicine, Shandong University, Jinan, China; ^4^University of Health and Rehabilitation Sciences, Qingdao, China

**Keywords:** stroke, repetitive transcranial magnetic stimulation, upper limb, motor function, meta-analysis

## Abstract

**Background:**

Repetitive transcranial magnetic stimulation (rTMS) is a promising therapy to promote recovery of the upper limb after stroke. According to the regulation of cortical excitability, rTMS can be divided into excitatory rTMS and inhibitory rTMS, and excitatory rTMS includes high-frequency rTMS (HF-rTMS) or intermittent theta-burst stimulation (iTBS). We aimed to evaluate the effects of excitatory rTMS over the ipsilesional hemisphere on upper limb motor recovery after stroke.

**Methods:**

Databases of PubMed, Embase, ISI Web of Science, and the Cochrane Library were searched for randomized controlled trials published before 31 December 2021. RCTs on the effects of HF-rTMS or iTBS on upper limb function in patients diagnosed with stroke were included. Two researchers independently screened the literature, extracted the data, and assessed quality. The meta-analysis was performed by using Review Manager Version 5.4 software.

**Results:**

Fifteen studies with 449 participants were included in this meta-analysis. This meta-analysis found that excitatory rTMS had significant efficacy on upper limb motor function (MD = 5.88, 95% CI, 3.32–8.43, *P* < 0.001), hand strength (SMD = 0.53, 95% CI, 0.04–1.01, *P* = 0.03), and hand dexterity (SMD = 0.76, 95% CI, 0.39–1.14, *P* < 0.001). Subgroup analyses based on different types of rTMS showed that both iTBS and HF-rTMS significantly promoted upper limb motor function (iTBS, *P* < 0.001; HF-rTMS, *P* < 0.001) and hand dexterity (iTBS, *P* = 0.01; HF-rTMS, *P* < 0.001) but not hand strength (iTBS, *P* = 0.07; HF-rTMS, *P* = 0.12). Further subgroup analysis based on the duration of illness demonstrated that applying excitatory rTMS during the first 3 months (<1 month, *P* = 0.01; 1–3 months, *P* = 0.001) after stroke brought significant improvement in upper limb motor function but not in the patients with a duration longer than 3 months (*P* = 0.06). We found that HF-rTMS significantly enhanced the motor evoked potential (MEP) amplitude of affected hemisphere (SMD = 0.82, 95% CI, 0.32–1.33, *P* = 0.001).

**Conclusion:**

Our study demonstrated that excitatory rTMS over the ipsilesional hemisphere could significantly improve upper limb motor function, hand strength, and hand dexterity in patients diagnosed with stroke. Both iTBS and HF-rTMS which could significantly promote upper limb motor function and hand dexterity, and excitatory rTMS were beneficial to upper limb motor function recovery only when applied in the first 3 months after stroke. HF-rTMS could significantly enhance the MEP amplitude of the affected hemisphere. High-quality and large-scale randomized controlled trials in the future are required to confirm our conclusions.

**Clinical Trial Registration:**

www.crd.york.ac.uk/prospero/, identifier: CRD42022312288.

## Introduction

Stroke is one of the major causes of death and disability in adults globally (1). According to the statistics, 55%−75% of post-stroke patients suffer from upper limb (UL) motor impairments (2). Despite receiving intensive rehabilitative therapies, many patients post-stroke retained motor dysfunction at variable degrees (3–5), which decreased health-related quality of life (6). Recently, many studies have suggested that repetitive transcranial magnetic stimulation (rTMS) may be a promising therapy for promoting recovery of the upper limb after stroke (7–10), possibly through modulating cortical excitability and inducing neural plasticity (11–15).

Repetitive TMS can be divided into excitatory rTMS and inhibitory rTMS according to their different regulatory effects on cortical excitability (16, 17). Excitatory rTMS includes high-frequency rTMS (HF-rTMS) or intermittent theta-burst stimulation (iTBS), which can increase cortical excitability, whereas inhibitory rTMS includes low-frequency rTMS (LF-rTMS) or continuous theta-burst stimulation (cTBS), which can suppress cortical excitability (18, 19). According to the interhemispheric inhibition (IHI) model, a theoretical model commonly used to guide the use of rTMS in motor rehabilitation after stroke, there is abnormally increased transcallosal inhibition from the contralateral to ipsilateral hemisphere after stroke, resulting in decreased cortex excitability of the ipsilateral hemisphere and increased cortex excitability of the contralateral hemisphere (20, 21). Therefore, excitatory rTMS is usually applied to the ipsilesional hemisphere, while inhibitory rTMS is applied to the contralateral hemisphere (22, 23).

Previous meta-analyses have studied the effects of rTMS, including excitatory rTMS and inhibitory rTMS (24, 25), or LF-rTMS alone on motor recovery in patients diagnosed with stroke (26), as well as the effects of treatment parameters and disease course on the efficacy of rTMS (27, 28), but so far, no in-depth systematic meta-analyses have examined the efficacy of excitatory rTMS over the ipsilesional hemisphere on motor recovery of upper limbs in patients diagnosed with stroke. The primary purpose of this systematic review was to investigate the effects of excitatory rTMS over the ipsilesional hemisphere on upper limb motor recovery after stroke. Since the efficacy of rTMS could be influenced by many factors, including the stimulating mode and the duration after stroke, we would also perform subgroup analyses based on the different types of rTMS (iTBS/HF-rTMS) and the duration post-stroke (<1 month/1–3 months/≥3 months) (27, 29).

## Methods

This systematic review was conducted according to the Preferred Reporting Items for Systematic Reviews and Meta-Analyses (PRISMA) guidelines (30). We registered the protocol in PROSPERO (registration no. CRD42022312288).

### Search Strategy

The databases of PubMed, Embase, ISI Web of Science, and the Cochrane Library were searched for the literature published up to December 31, 2021. We used the key terms “stroke,” “transcranial magnetic stimulation,” “upper limb function,” or their synonyms. The detailed search strategy is illustrated in Supplementary Table 1.

### Selection of Studies

The relevant articles were searched using the PICO principle, followed by screening on the basis of the inclusion and exclusion criteria. The inclusion criteria were as follows: (1) population: adult patients (≥18 years) diagnosed with stroke and suffering from upper extremity motor dysfunction; (2) interventions: HF-rTMS or iTBS over the ipsilesional hemisphere; (3) control: sham stimulation or conventional rehabilitation; (4) outcome: measures that evaluated the motor function of the upper limb or cortical excitability; (5) study type: parallel randomized controlled trials (RCTs); and (6) language is limited to English. The following exclusion criteria were applied: (1) rTMS was part of a coupling/priming protocol or it was bilateral; (2) the study received a PEDro scale (Physiotherapy Evidence Database from the Center for Evidence-Based Physiotherapy of The George Institute for Global Health) rating of “poor”, defined as 3 or less (see below, quality assessment); and (3) information required to perform a meta-analysis (e.g., mean scores, standard deviations) was missing after attempts to contact the corresponding author. The two researchers (ZT and KH) independently reviewed the titles and abstracts, when necessary, and read the entire text of the articles to determine whether they should be included in the study. If there was a disagreement, the two researchers discussed and reached a consensus with a third reviewer (RW).

### Quality Assessment

The PEDro scale was applied to evaluate the methodological quality of the studies (31–33). There are 11 items on the scale, with a maximum score of 10 (9–10: excellent; 6–8: good; 4–5: fair; and ≤3: poor) (34, 35). The bias risk assessment tool (Cochrane5.1.0 version) was used to appraise the risk of bias (36), including selection bias, performance bias, detection bias, attrition bias, reporting bias, and other biases. Each domain was rated as “low,” “high,” or “unclear” for each study. The methodological quality and risk of bias were rated independently by two reviewers (ZT and KH). Any disagreements were resolved by contacting a third reviewer (YZ).

### Data Extraction

Two researchers independently screened the literature, extracted the data, and cross-checked them (ZT and KH). In case of disagreement, it was discussed or reviewed by the third researcher (RW) until a consensus was reached. For each study, the following information was extracted: number of subjects, demographic characteristics of the patients, disease characteristics, rTMS protocol, additional intervention, control condition, outcome measures, mean differences, and standard deviations (SDs) of the change scores or means and SDs of the scores after intervention. If the results were only graphically presented, we used the software GetData Graph Digitizer 2.20 to extract the desired data, as the previous researchers did (26).

### Data Synthesis and Analysis

The upper limb motor recovery outcome of patients diagnosed with stroke was divided into three categories: upper limb motor function, hand strength, and hand dexterity. The upper extremity Fugl-Meyer Assessment (UE-FMA) was used to evaluate upper limb motor function. The results of pinch force and grip force were used to evaluate hand strength. The results of the action research arm test (ARAT), Box and Block Test (BBT), Jebsen-Taylor test (JTT), Wolf motor function test (WMFT), and nine-hole peg test (NHPT) were pooled to evaluate hand dexterity (37). In addition, the motor evoked potential (MEP) amplitude was used to assess cortical excitability (38). The Review Manager Version 5.4 was used for all analyses (39). When different scales were used for outcome measures and the outcome was a continuous variable, effect size would be reported as standardized mean differences (SMD) with 95% confidence intervals (CI) instead of mean differences (MD). Cochran's Q-test and the I^2^ statistic were performed to assess the heterogeneity of the effect sizes. If *I*^2^ was >50% and *P* < 0.1, a random-effects model was applied; otherwise, the fixed-effects model was used for data analysis (40). The statistical signific ance value was set as *P* < 0.05.

## Results

### Study Selection

**Figure 1:**
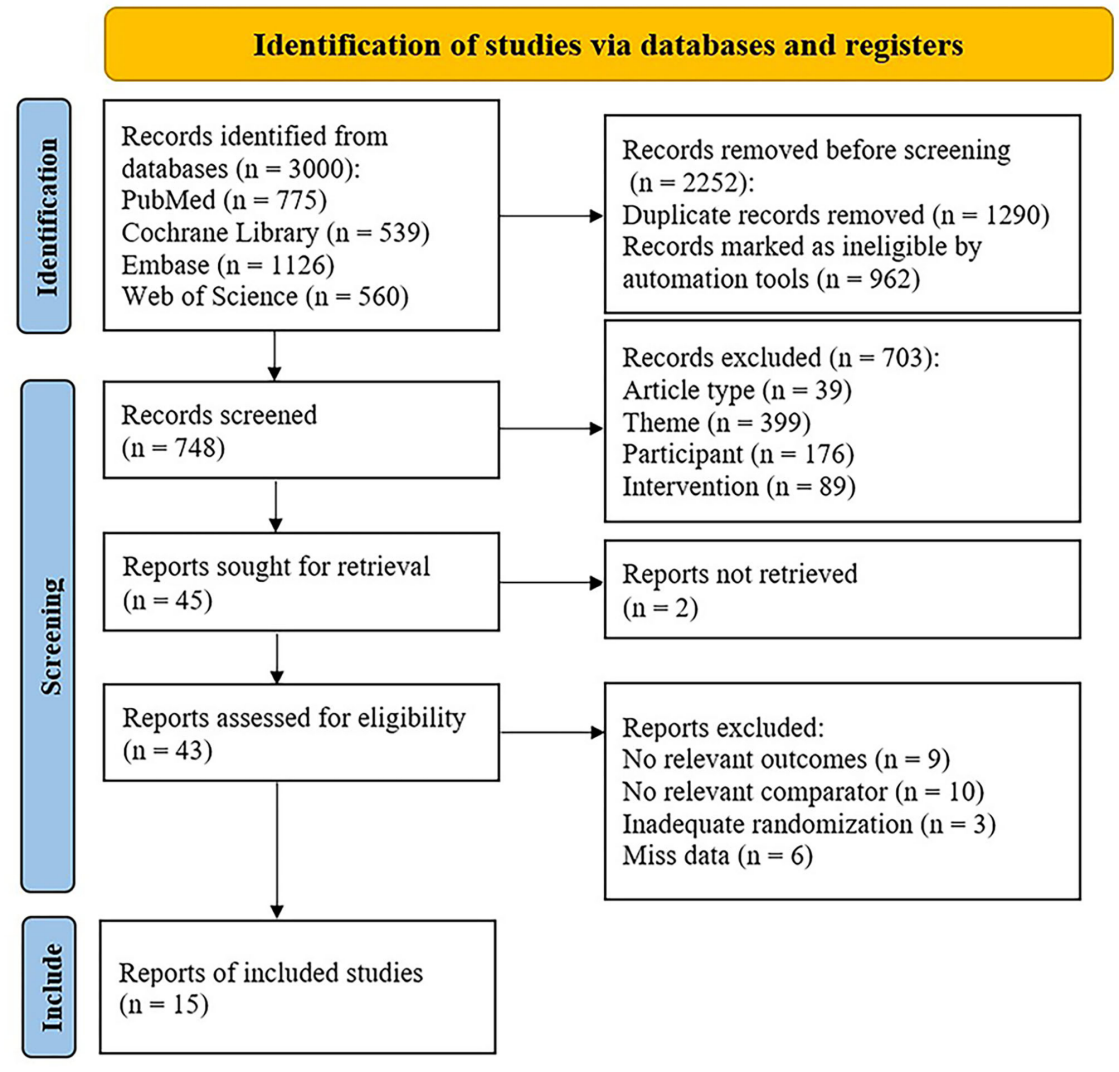
Flow diagram of the study selection.

Of 3,000 relevant articles identified in the initial database search, 2,985 articles were excluded after screening the titles and abstracts and removing duplicates. Finally, 15 studies were included in this meta-analysis, involving a total of 449 subjects (41–55). The literature selection is presented in Figure 1.

### Study Characteristics

The characteristics of included studies are given in detail in Table 1. All studies were designed as randomized controlled parallel studies. Subject numbers of the RCTs included ranged from 12 (42) to 85 (43) patients, with a mean age ranging from 48.95 (53) to 71 years (45). The duration of stroke onset in the included subjects ranged from 3.8 days (49) to 20 months (45). Among them, the duration of subjects in six studies was <1 month (41–43, 46, 49, 52), the duration of subjects in three studies was 1–3 months (47, 48, 55), and the duration of subjects in six studies was more than 3 months (44, 45, 50, 51, 53, 54). All of the included studies applied HF-rTMS or iTBS over the ipsilesional M1 (primary motor cortex). Four studies used iTBS (42, 45, 51, 53) while others used HF-rTMS (41, 43, 44, 46–50, 52, 54, 55). Only one study used round toil, and the rest used a figure of eight coils. The number of sessions varied from 5 (41, 43, 46, 52) to 20 (44). In addition, only two studies used conventional rehabilitation programs as a control condition (54, 55), and the others used sham stimulation (41–53), such as sham coils or tilted coils.

**Table 1:** Characteristics of the included studies.

**References**	**Sample size (E/C)**	**Age (year) (E/C)**	**Gender (M/F)**	**Onset time (E/C)**	**Hemiparesis (R/L)**	**Stroke type (I/H)**	**TMS protocol**	**Control condition**	**Outcome measures**	**Additional intervention**
Ackerley et al. (45)	9/9	61/71	12/6	20 months/18 months	6/12	NR	M1, iTBS, 90% AMT, 600 pulses, 10 sessions	Sham coil	UE-FMA, ARAT	Conventional rehabilitation
Chen et al. (51)	11/11	52.9/52.6	14/8	≥6 months	15/7	5/17	M1, iTBS, 80% AMT, 600 pulses, 10 sessions	Tilted coil, 60% AMT	UE-FMA, ARAT, BBT	Conventional rehabilitation
Chen et al. (53)	12/11	54.36/48.95	18/5	5.01/7.99months	14/9	8/15	M1, iTBS, 80% AMT, 600 pulses, 15 sessions	Tilted coil, 60% AMT	UE-FMA, BBT, ARAT, NHPT	Virtual reality-based cycling training
Chervyakov et al. (50)	13/10	58.6/61.4	15/8	5.8/7.9 months	8/15	NR	M1, HF-rTMS, 10Hz, 80%RMT, 2,000 pulses, 10 sessions	Coil disconnected	UE-FMA	Physical therapy
Du et al. (46)	20/19	56.78/53.6	29/17	7/8 days	21/25	NR	M1, HF-rTMS, 3Hz, 80%−90% RMT, 1,200 pulses, 5 sessions	Tilted coil	UE-FMA, MEP	Conventional rehabilitation
Du et al. (52)	15/13	54/56	30/10	5/4 days	25/15	40/0	M1, HF-rTMS, 10Hz, 100%RMT, 1,200 pulses, 5 sessions	Tilted coil	UE-FMA, MEP	Conventional rehabilitation
Guan et al. (49)	21/21	59.7/57.4	30/12	3.8/4.8 days	23/19	42/0	M1, HF-rTMS, 5Hz, 120%RMT, 1,000 pulses, 10 sessions	Tilted coil	RMT, UE-FMA	Motor rehabilitative training
Moslemi et al. (54)	10/10	50.50/53.90	11/9	3.00/3.20 months	9/11	NR	M1, HF-rTMS, 20Hz, 90%RMT, 2,000 pulses, 10 sessions	Rehabilitation program	UE-FMA, BBT, GS, PS	Rehabilitation program
Hosomi et al. (47)	18/21	62.4/63.2	23/16	46.1/45.1 days	15/24	24/15	M1, HF-rTMS, 5Hz, 90%RMT, 500 pulses, 10 sessions	Tilted coil	GS, FMA	Conventional rehabilitation
Hsu et al. (42)	6/6	56.8/62.3	8/4	22.0/20.8 days	4/8	12/0	M1, iTBS, 80%AMT, 1,200 pulses, 10 sessions	Tilted coil	UE-FMA, ARAT, MEP	Conventional rehabilitation
Khedr et al. (41)	12/12	59.0/60.0	12/12	17.2/17.7 days	8/16	24/0	M1, HF-rTMS, 3Hz, 130% RMT, 900 pulses, 5 sessions	Tilted coil	MEP, MRC, PPT	Conventional rehabilitation
Kim et al. (44)	16/15	62.40/61.80	14/17	3.70/4.89 months	NR	NR	M1, HF-rTMS, 10Hz, 80% AMT, 500 pulses, 20 sessions	0%RMT	MEP	Task oriented training
Li et al. (48)	43/42	54.00/53.13	57/28	1.36/1.58 months	39/46	85/0	M1, HF-rTMS, 10Hz, 80% MT, 1,350 pulses, 10 sessions	Sham coil	UE-FMA, WMFT, MEP	Conventional rehabilitation
Sasaki et al. (43)	9/9	65.7/63.0	12/6	18.4/15.4 days	8/10	8/10	M1, HF-rTMS, 10Hz, 90% RMT, 1,000 pulses, 5 sessions	Tilted coil	GS	NR
Yang et al. (55)	12/13	64/64	18/7	64/75 days	NR	20/5	M1, HF-rTMS, 5Hz, 100% RMT, 750 pulses, 10 sessions	Hand grip training	UE-FMA, GS, MEP, JTT	Conventional Rehabilitation, hand grip training

*E, experimental group; C, control group; M, male; F, female; R, right; L, left; I, ischemic; H, hemorrhagic; NR, not reported; AMT, active motor threshold; RMT, resting motor threshold; MT, motor threshold; UE-FMA, upper extremity Fugl-Meyer Assessment; ARAT, Action Research Arm Test; BBT, Box and Block Test; NHPT, nine-hole peg test; MEP, motor evoked potential; GS, grip strength; PS, pinch strength; WMFT, Wolf motor function test; JTT, Jebsen-Taylor test*.

### Quality Assessment

**Figure 2:**
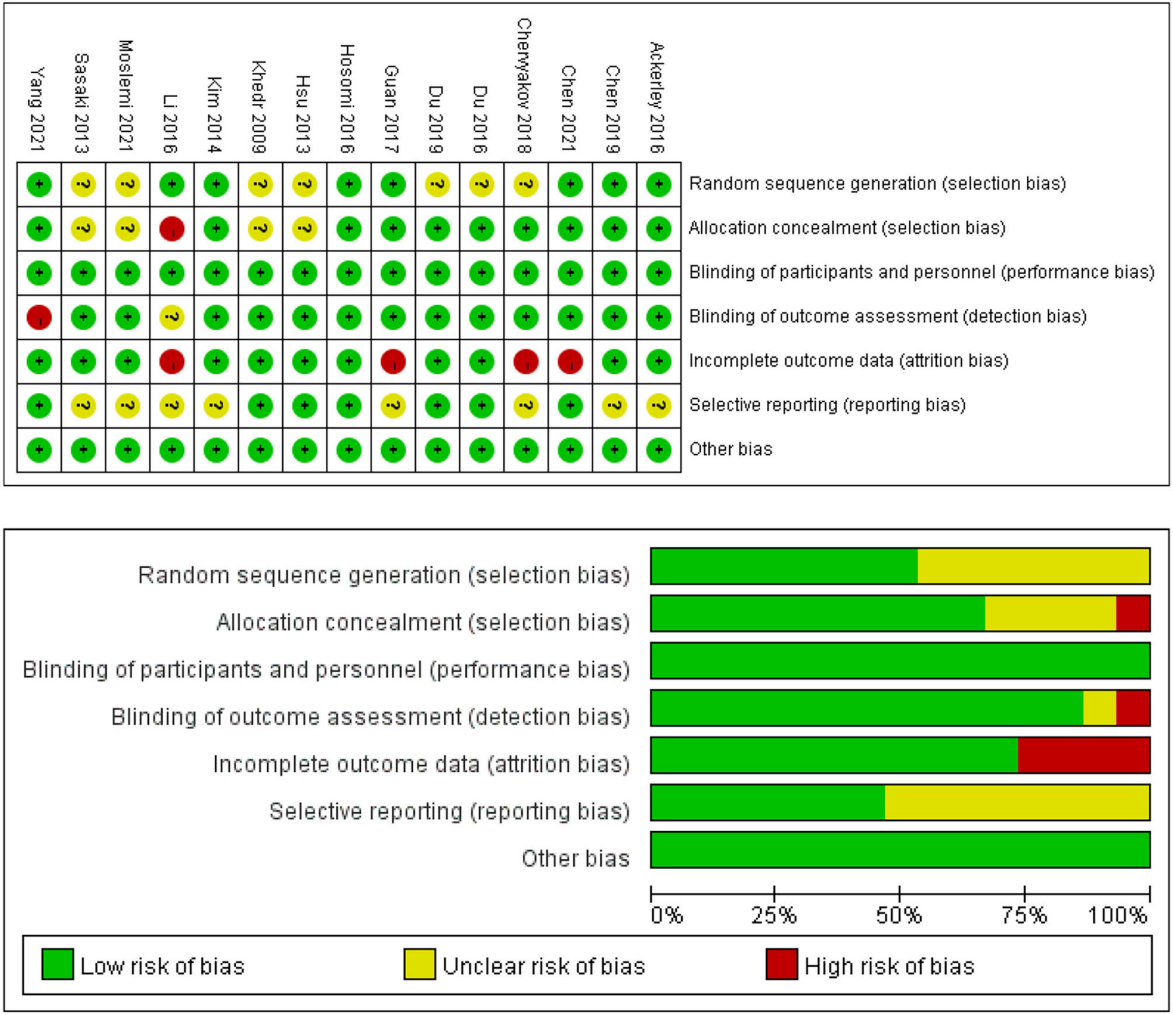
Risk of bias summary and graph for each risk of bias item presented as percentages across all included studies.

The PEDro scores of the included studies ranged from 6 (48) to 10 (44–46, 49, 52). Of all included studies, 10 studies were of excellent quality (41, 44–47, 49–53), and five studies were of good quality (42, 43, 48, 54, 55). No studies were assessed as fair quality or poor quality. The detailed results of the methodological quality assessment are shown in Table 2. The risk of bias for all included studies was evaluated with the Cochrane Risk of Bias Tool, and the results are shown in Figure 2. Eight studies described a random sequence generation and were evaluated as low risk (44, 45, 47–49, 51, 53, 55). Ten studies demonstrated a low risk of bias through allocation concealment (44–47, 49–53, 55). Thirteen studies were explicitly assessor-blinded and were classified as at low risk of detection bias (41–47, 49–54). Four studies demonstrated a high risk of bias due to incomplete outcome data (37, 48–50, 53). As for reporting bias, eight studies were classified as at unclear risk (43–45, 48–51, 54). There was no selective performance and no other bias in all studies.

**Table 2:** Risk of bias assessment according to the Physiotherapy Evidence Database scale.

**Study**	**Criteria**											**Total**	**Quality**
	**1**	**2**	**3**	**4**	**5**	**6**	**7**	**8**	**9**	**10**	**11**		
Ackerley et al. (45)	1	1	1	1	1	1	1	1	1	1	1	10	Excellent
Chen et al. (51)	1	1	1	1	1	0	1	1	1	1	1	9	Excellent
Chen et al. (53)	1	1	1	1	1	0	1	1	1	1	1	9	Excellent
Chervyakov et al. (50)	1	1	1	1	1	0	1	1	1	1	1	9	Excellent
Du et al. (46)	1	1	1	1	1	1	1	1	1	1	1	10	Excellent
Du et al. (52)	1	1	1	1	1	1	1	1	1	1	1	10	Excellent
Guan et al. (49)	1	1	1	1	1	1	1	1	1	1	1	10	Excellent
Moslemi et al. (54)	1	1	0	1	1	0	1	1	1	1	1	8	Good
Hosomi et al. (47)	1	1	1	1	1	0	1	1	1	1	1	9	Excellent
Hsu et al. (42)	1	1	0	1	1	1	1	1	1	1	1	9	Good
Khedr et al. (41)	1	1	1	1	1	0	1	1	1	1	1	9	Excellent
Kim et al. (44)	1	1	1	1	1	1	1	1	1	1	1	10	Excellent
Li et al. (48)	1	1	0	1	1	0	0	0	1	1	1	6	Good
Sasaki et al. (43)	1	1	0	1	1	0	1	1	1	1	1	8	Good
Yang et al. (55)	1	1	1	1	1	0	0	1	1	1	1	8	Good

*Criteria numbers: 1, eligibility criteria and source of participants; 2, random allocation; 3, concealed allocation; 4, baseline comparability; 5, participant blinding; 6, therapist blinding; 7, assessor blinding; 8, outcome obtained in more than 85% of the subjects; 9, intention-to-treat analysis; 10, between-group comparison; 11, point estimates and variability*.

### Effects on Upper Limb Motor Function

**Figure 3:**
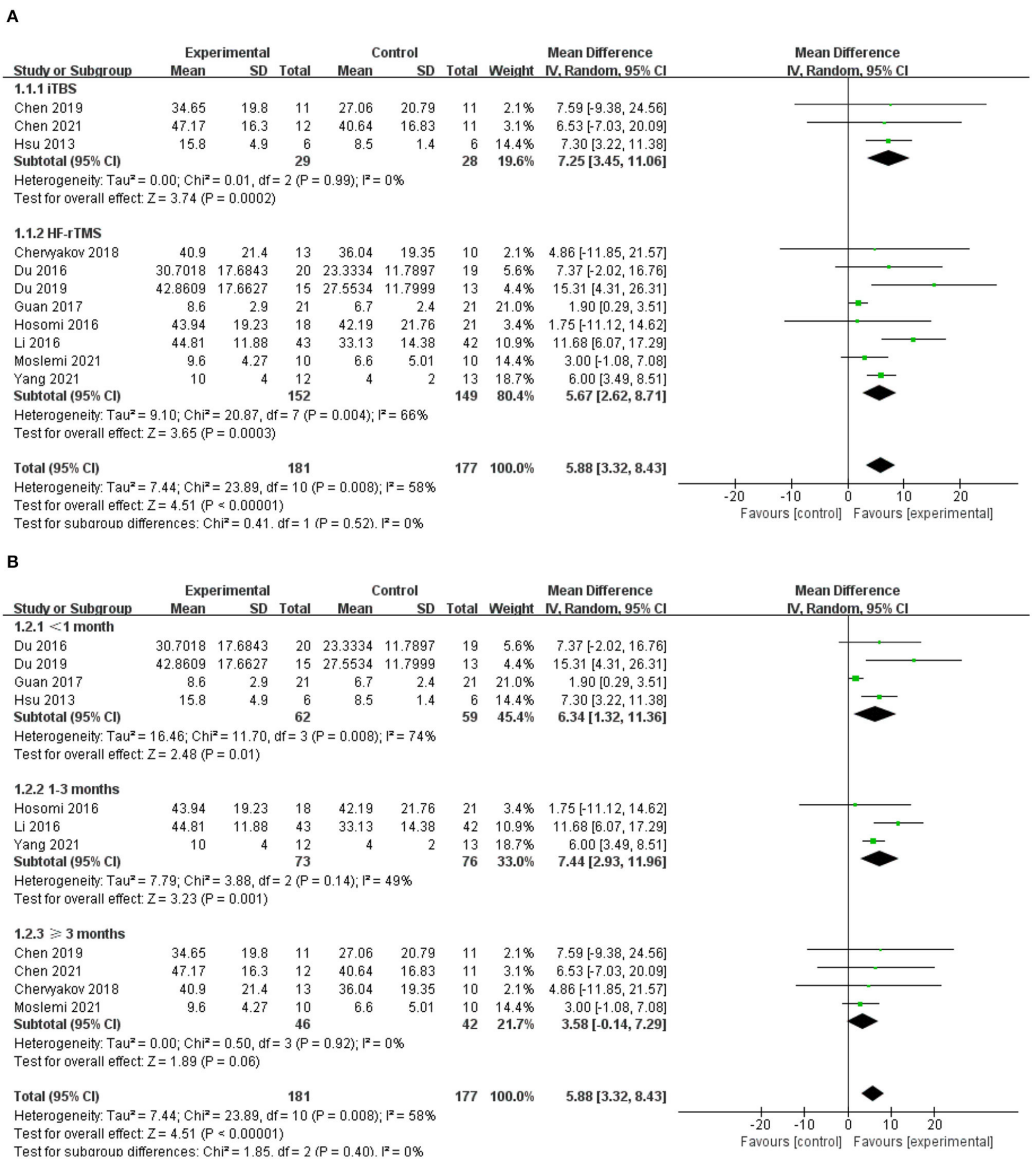
**(A)** Forest plot from the meta-analysis of excitatory rTMS on upper limb motor function showing estimates of effect size (MD) with 95% confidence intervals: subgroup analysis based on different types of rTMS. **(B)** Forest plot from the meta- analysis of excitatory rTMS on upper limb motor function showing estimates of effect size (MD) with 95% confidence intervals: subgroup analysis based on the duration post-stroke.

Eleven of the included studies used UE-FMA to evaluate upper limb motor function in patients diagnosed with stroke, with a total of 181 subjects (42, 46–55). The results showed that the experimental group was significantly better than the control group in improving UE-FMA scores (MD = 5.88; 95% CI, 3.32 to 8.43; *P* < 0.001; *I*^2^ = 58%, Figure 3A). Further subgroup analysis based on different types of rTMS (iTBS/HF-rTMS) over the ipsilesional M1 showed insignificant differences among groups (*P* = 0.52; *I*^2^ = 0%, Figure 3A). Both iTBS and HF-rTMS brought significant improvement of UE-FMA scores (iTBS, MD = 7.25; 95% CI, 3.45 to 11.06; *P* < 0.001, vs. HF-rTMS, MD = 5.67; 95% CI, 2.62 to 8.71; *P* < 0.001, Figure 3A). Meanwhile, we performed the subgroup analysis according to the duration post-stroke, and the results suggested that excitatory rTMS had no significant effects on upper limb motor function in patients with a duration of disease longer than 3 months (MD = 3.58; 95% CI, −0.14 to 7.29; *P* = 0.06; *I*^2^ = 0%, Figure 3B).

### Effects on Hand Strength

**Figure 4:**
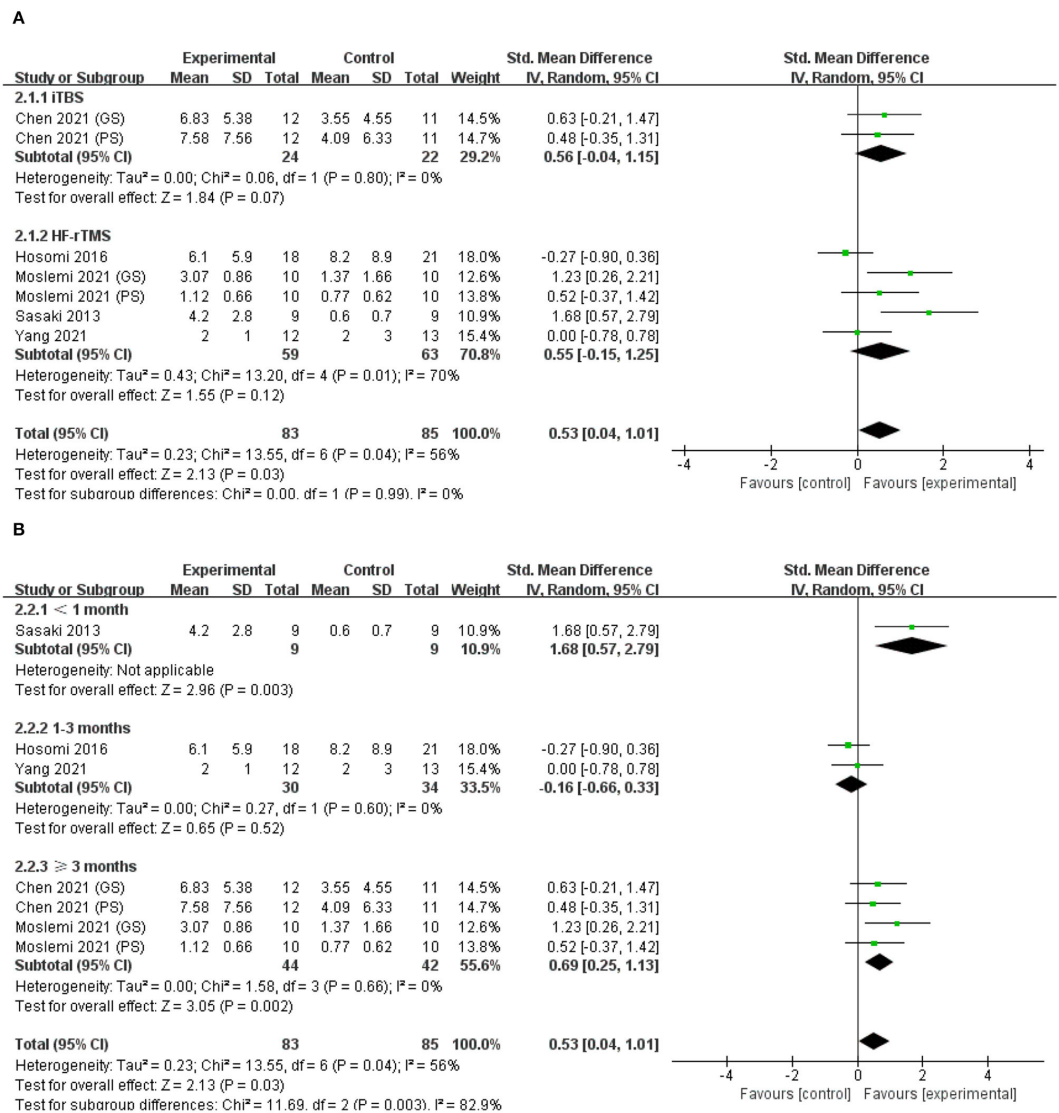
**(A)** Forest plot from the meta-analysis of excitatory rTMS on hand strength showing estimates of effect size (SMD) with 95% confidence intervals: subgroup analysis based on different types of rTMS. **(B)** Forest plot from the meta- analysis of excitatory rTMS on hand strength showing estimates of effect size (SMD) with 95% confidence intervals: subgroup analysis based on the duration post-stroke.

Five studies assessed grip strength (43, 47, 53–55), two of which also evaluated pinch strength (53, 54). The meta-analysis in a random-effects model showed significant difference in hand strength for real rTMS relative to control condition (SMD = 0.53; 95% CI, 0.04 to 1.01; *P* = 0.03; *I*^2^ = 56%, Figure 4A). Subgroup analysis based on different types of rTMS (iTBS/HF-rTMS) also suggested no significant difference between groups (*P* = 0.99; *I*^2^ = 0%, Figure 4A). However, neither iTBS nor HF-rTMS was significantly better than the control group in improving hand strength (iTBS, SMD = 0.56; 95% CI, −0.04 to 1.15; *P* = 0.07, vs. HF-rTMS, SMD = 0.55; 95% CI, −0.15 to 1.25; *P* = 0.12, Figure 4A). Further subgroup analysis based on the duration of illness indicated that there was significant difference among groups (<1 month, SMD = 1.68; 95% CI, 0.57 to 2.79; *P* = 0.003, vs. 1–3 months, SMD = −0.16; 95% CI, −0.66 to 0.33; *P* = 0.52, vs. ≥ 3 months, SMD = 0.69; 95% CI, −0.25 to 1.13; *P* = 0.002, Figure 4B).

### Effects on Hand Dexterity

**Figure 5:**
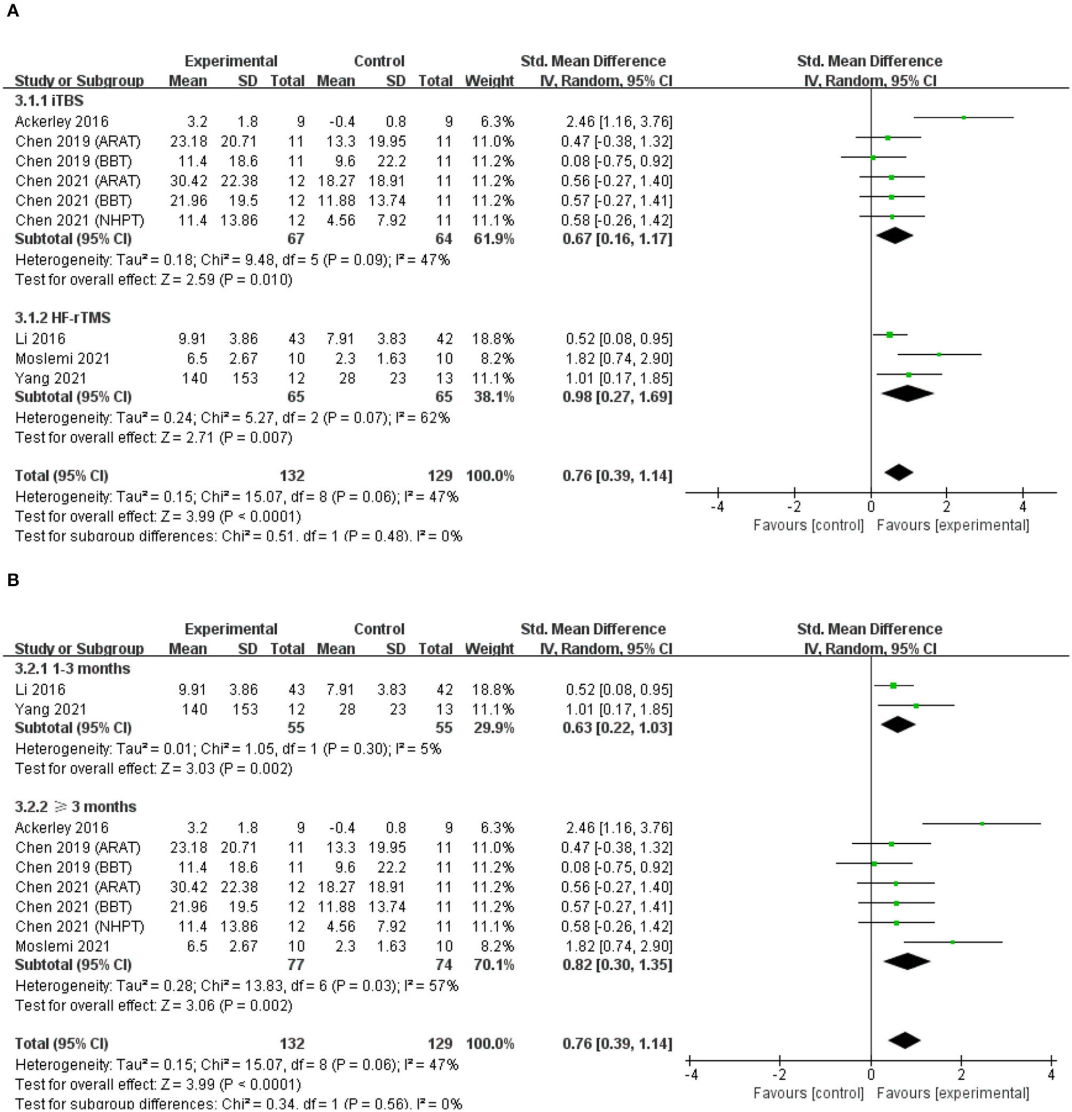
**(A)** Forest plot from the meta-analysis of excitatory rTMS on hand dexterity showing estimates of effect size (SMD) with 95% confidence intervals: subgroup analysis based on different types of rTMS. **(B)** Forest plot from the meta- analysis of excitatory rTMS on hand dexterity showing estimates of effect size (SMD) with 95% confidence intervals: subgroup analysis based on the duration post-stroke.

Six studies were pooled to investigate the effects of excitatory rTMS over the ipsilesional hemisphere on hand dexterity (45, 48, 51, 53–55). The results for hand dexterity indicated that there were significant differences between the experimental group and the control group (SMD = 0.76; 95% CI, 0.39 to 1.14; *P* < 0.001; *I*^2^ = 47%, Figure 5A). There was no significant difference in subgroup analysis based on different types of rTMS (iTBS/HF-rTMS; iTBS, SMD = 0.67; 95% CI, 0.16 to 1.17; *P* = 0.01, vs. HF-rTMS, SMD = 0.98; 95% CI, 0.27 to 1.69; *P* = 0.007, Figure 5A). When grouped by duration of disease, the subgroup analysis showed insignificant difference between groups (1–3 months, SMD = 0.63; 95% CI, 0.22 to 1.03; *P* = 0.002, vs. ≥3 months, SMD = 0.82; 95% CI, 0.30 to 1.35; *P* = 0.002, Figure 5B).

### Effects on Cortical Excitability

**Figure 6:**
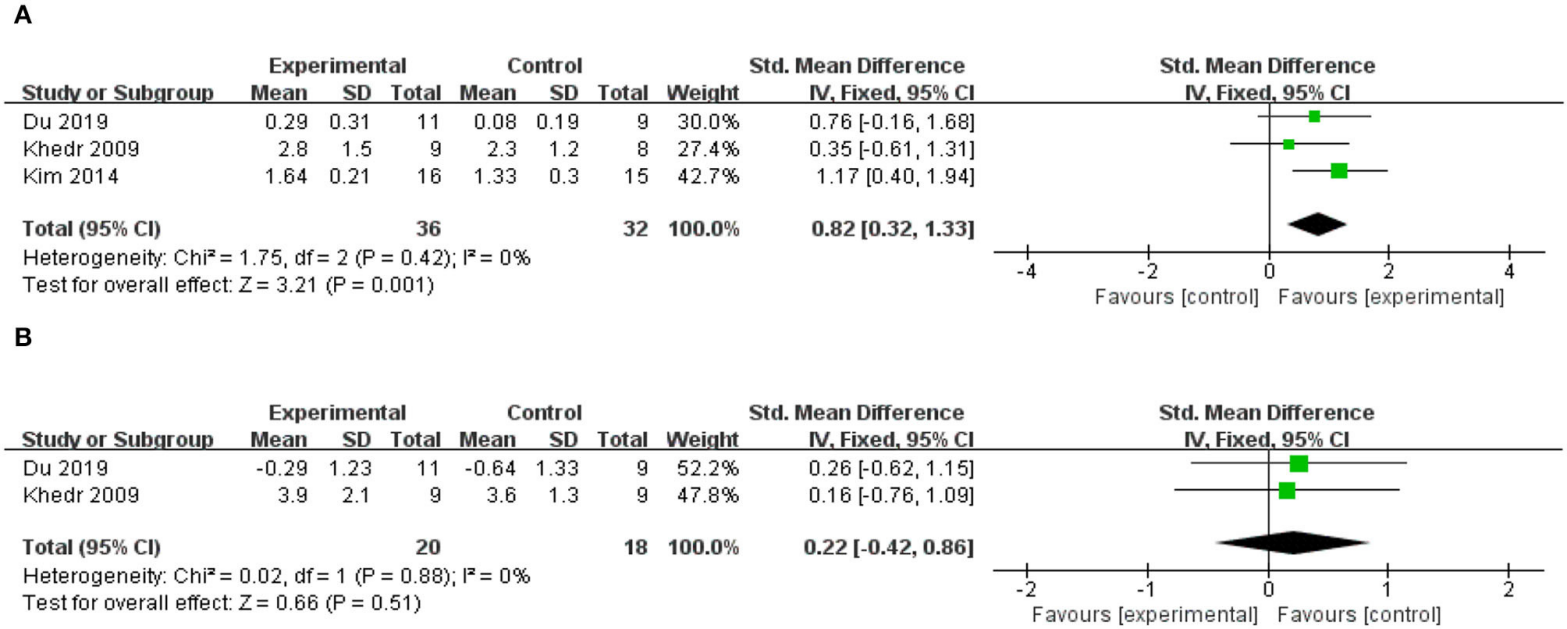
**(A)** Forest plot from the meta-analysis of excitatory rTMS on MEP amplitude in the affected hemisphere showing estimates of effect size (SMD) with 95% confidence intervals. **(B)** Forest plot from the meta-analysis of excitatory rTMS on MEP amplitude in the unaffected hemisphere showing estimates of effect size (SMD) with 95% confidence intervals.

Three included studies reported changes in MEP amplitude in the affected hemisphere (41, 44, 52), all of which applied HF-rTMS over the ipsilesional hemisphere, and two of these studies reported changes in MEP amplitudes in the unaffected hemispheres at the same time (41, 52). The meta-analysis results revealed that the rTMS-treated group exhibited higher levels of improvement than the control group did in MEP amplitude of affected hemisphere (SMD = 0.82; 95% CI, 0.32 to 1.33; *P* = 0.001; *I*^2^ = 0%, Figure 6A). Contrarily, an insignificant difference between groups was observed in MEP amplitude of unaffected hemisphere (SMD = 0.22; 95% CI, −0.42 to 0.86; *P* = 0.51; *I*^2^ = 0%, Figure 6B). Due to the small number of included studies, we did not conduct a subgroup analysis.

#### Adverse Events

Of the 15 studies included in this review, only one study (44) did not mention about having adverse events or not. Nine studies reported no adverse events (41, 43, 45, 47, 49, 51, 53–55), four studies reported transient headaches (42, 46, 50, 52), and two reported tingling sensations on the head (42, 52). Li et al. (48) reported that a few patients experienced numbness in the scalp and facial muscles, which were bearable, and disappeared when stimulation stopped. Only one study reported serious adverse events such as seizures, increased paroxysmal or newly emerged epileptiform EEG activity, and lower extremity deep vein thrombosis and thrombus flotation. What called for special attention was that the researchers also pointed out that seizures could have been prevented by excluding these patients, as the epileptiform signs were seen on EEG records during the initial screening (50).

## Discussion

To our knowledge, this is the first meta-analysis performed to explore the effects of excitatory rTMS over the ipsilesional hemisphere on upper limb motor recovery after stroke. The results provided evidence that both iTBS and HF-rTMS over the ipsilesional primary motor cortex significantly improved upper limb motor function, hand strength, and hand dexterity in patients diagnosed with stroke, and our study found HF-rTMS enhanced MEP amplitude of the affected hemisphere.

It was worth noting that the evidence level may be decreased because of the risks of bias identified in the included randomized controlled trials. The main sources of bias in all included randomized controlled trials were selection bias, detection bias, and attrition bias. In some of the included studies, the methods of concealment of allocation and blinding to the assessors were unclearly described. Additionally, some of the included randomized controlled trials reported incomplete outcome data. More excellent-quality randomized controlled trials should be performed to provide further evidence regarding the benefits of rTMS for improving upper limb motor function after stroke.

Since it is difficult to compare all the outcomes of the studies, there are different motor scales to measure upper limb function (56), and different motor scales measured the domains differently; the motor outcomes were divided into three categories: upper limb motor function, hand strength, and hand dexterity, similar to the previous studies (26, 37). The UE-FMA, a reliable and valid scale (57–59), is widely used for the assessment of upper limb motor function for patients after stroke. Although previous meta-analyses and systematic reviews concluded that rTMS was beneficial for motor recovery after stroke (60–63), these reviews did not examine the role of excitatory rTMS alone or the effect on upper limb function alone. This meta-analysis made up for it by demonstrating that excitatory rTMS significantly improved upper limb motor function represented by the UE-FMA scores. Our results were inconsistent with the results of a previous meta-analysis, which suggested that a combination of rTMS and upper limb training did not have a stronger effect on upper limb function than upper limb training alone (64). It should be noted that all of the included studies added additional upper limb rehabilitation training to rTMS, and thirteen studies used sham stimulation as a control condition. A new guideline on the therapeutic use of rTMS showed that the current level of evidence was in favor of a probable beneficial impact of ipsilesional HF-rTMS of M1 in the post-acute phase of stroke for promoting upper motor function recovery (Level B) (65), and our results supported that excitatory rTMS could be beneficial to the recovery of upper limb motor function in patients with a duration of disease <3 months. However, subgroup analysis based on the duration post-stroke demonstrated that applying excitatory rTMS over ipsilesional M1 had no significant effects on upper limb motor function in patients with a duration of disease longer than 3 months. It could not be ignored that the model of interhemispheric inhibition has been largely challenged in recent years. Another contradictory theoretical model, the vicariation model, holds that activity in the unaffected hemisphere can contribute to functional recovery after stroke, and inhibition of the excitability of the unaffected hemisphere will obstruct the functional recovery after stroke (66), as confirmed by Wang et al. (67). Di Pino et al. (22) thought that existing models were insufficient to explain the recovery of all patients and proposed a new theoretical model–the bimodal balance–recovery model. This model introduced the concept of “structural reserve,” which determined whether the interhemispheric imbalance model was superior to the compensatory model. Since it is highly likely that the interhemispheric inhibition model is more suitable for patients with subcortical, chronic, and rather mild impairment (68), we hypothesize that inhibitory stimulation over the contralateral hemisphere may be more effective in improving upper limb motor function in patients diagnosed with stroke with a duration longer than 3 months, but unfortunately, few studies have compared the efficacy of excitatory stimulation and inhibitory stimulation at the chronic stage of stroke.

Although our results found excitatory rTMS significantly enhanced hand strength, which was consistent with the conclusion of a previous meta-analysis that noninvasive brain stimulation successfully improved paretic limb force production capabilities (69), we found neither iTBS nor HF-rTMS to be significantly better than the control group in enhancing hand strength. Due to the existence of heterogeneity and the small number of included studies, we need to treat the results with caution. Meanwhile, our results suggested that excitatory rTMS was able to promote the improvement of hand dexterity after stroke, which was consistent with the conclusion of the previous meta-analysis conducted by O'Brien et al. (70). The authors found that noninvasive brain stimulation had a significant effect on the improvement of dexterity in chronic stroke stages, probably through motor learning mechanisms. Our subgroup analysis also showed that excitatory rTMS significantly improved hand dexterity of patients diagnosed with stroke with a duration of disease longer than 1 month. Studies with a disease duration of less than a month were not included, perhaps because most patients mainly achieved recovery of proximal upper limb function during this period. The recovery of fine hand movement has always been considered a difficulty in stroke rehabilitation, and our results undoubtedly provided evidence for the clinical application of excitatory rTMS.

It should be noted that our results were encouraging as they showed that both iTBS and HF-rTMS could significantly promote upper limb motor function recovery and hand dexterity. In recent years, compared with rTMS, TBS, a very potential noninvasive brain stimulation technology, has the advantages of shorter stimulation time and lower stimulation intensity (18, 71), and some researchers have indicated that TBS yielded comparable or even greater MEPs with longer-lasting effects than conventional rTMS (72–74), so it has attracted extensive attention. Although several studies have compared the efficacy of rTMS and TBS for motor recovery after stroke (8–10), these studies unfortunately have not reached a consistent conclusion, and few studies have compared HF-rTMS and iTBS. Thus, our results provided evidence for the clinical application of iTBS. In the future, iTBS may be more widely used in clinical practice because of its saving time and good efficacy. However, there are few studies about iTBS in the treatment of upper limb motor dysfunction after stroke, and only four articles were included in this meta-analysis, which may affect the reliability of our conclusions. We still need more randomized controlled studies to confirm our conclusions.

Another encouraging finding was that HF-rTMS induced a highly significant enhancing effect on the MEP amplitude of the ipsilesional hemisphere. The previous studies showed that rTMS could modulate cortical excitability (75, 76), and our study confirmed this again. Similar to our results, a recent meta-analysis performed by Bai et al. (77) found that HF-rTMS enhanced the cortical excitability of the affected M1 and iTBS, which also showed superior effects in rebalancing bilateral excitability. However, we found no significant inhibitory effect of HF-rTMS on contralateral M1. Thus, we believe that the possible mechanism of HF-rTMS promoting exercise recovery is mainly to increase the excitability of affected side M1, while inhibitory rTMS not only suppresses the cortical excitability of the unaffected M1 but also simultaneously enhances the cortical excitability of the affected M1. Unfortunately, in this study, we did not include relevant studies that explored the regulation of cortical excitability by iTBS.

### Limitations

This meta-analysis was not free from limitations. First, the results should be interpreted with caution because of the bias in some included studies, the fact that only one study represented a subgroup in some subgroup analyses and the fact that the effect sizes of treatment were often based on a mixture of change scores and final scores. Second, several variables, such as age, sex, side of onset, the severity of motor deficit, session numbers, stimulus intensity, and the number of pulses, could confound the results and must be acknowledged. Third, excitatory rTMS may have after-effects, but our study only looked at immediate effects but not long-term effects.

## Conclusions

The current study systematically reviewed existing research investigating the effects of excitatory rTMS in promoting upper limb motor recovery after stroke. Our results demonstrated that excitatory rTMS over the ipsilesional hemisphere could significantly improve upper limb motor function, hand strength, and hand dexterity in patients diagnosed with stroke. Both iTBS and HF-rTMS could significantly promote upper limb motor function and hand dexterity, and excitatory rTMS were beneficial to upper limb motor function recovery only when applied in the first 3 months after stroke. HF-rTMS may promote motor recovery by enhancing the excitability of M1 on the affected side. High-quality and large-scale randomized controlled trials for the future are required to confirm our conclusions.

## Data Availability Statement

The raw data supporting the conclusions of this article will be made available by the authors, without undue reservation.

## Author Contributions

HZ contributed to the conception and design of the study and provided guidance during the whole process of the study. ZT, KH, RW, and YZ contributed to study searching and screening, quality assessment, data extraction, and data analysis. ZT wrote the manuscript, and the other authors reviewed the manuscript. All authors approved the final version of the manuscript for submission.

## Funding

This work was supported by the special scientific research project for health development in the capital (No.2020-1-6011) and the general project of the China Rehabilitation Research Center (No. 2021ZX-18).

## Conflict of Interest

The authors declare that the research was conducted in the absence of any commercial or financial relationships that could be construed as a potential conflict of interest.

## Publisher's Note

All claims expressed in this article are solely those of the authors and do not necessarily represent those of their affiliated organizations, or those of the publisher, the editors and the reviewers. Any product that may be evaluated in this article, or claim that may be made by its manufacturer, is not guaranteed or endorsed by the publisher.
