# Supplementary material for: Excitatory Repetitive Transcranial Magnetic Stimulation Over the Ipsilesional Hemisphere for Upper Limb Motor Function After Stroke: A Systematic Review and Meta-Analysis
Source: Front Neurol. 2022 Jun 20;13:918597. doi: 10.3389/fneur.2022.918597 (PMC9251503; doi:10.3389/fneur.2022.918597)
Supplement: Supplementary file 1 [file Table_1.DOCX]

Supplementary Material

Pubmed

(("Transcranial Magnetic Stimulation"[Mesh] OR

TMS[Title/Abstract] OR

Repetitive transcranial magnetic stimulat*[Title/Abstract] OR

rTMS[Title/Abstract] OR

TBS[Title/Abstract] OR

iTBS[Title/Abstract] OR

theta burst stimulation [Title/Abstract] OR

θ burst stimulation [Title/Abstract])

AND

("Stroke"[Mesh] OR "Brain Ischemia"[Mesh] OR

"Intracranial Hemorrhages"[Mesh] OR

stroke[Title/Abstract] OR

CVA[Title/Abstract] OR

CVAs[Title/Abstract] OR

cerebrovascular accident*[Title/Abstract] OR

cerebral ischemia*[Title/Abstract] OR

cerebral ischaemia*[Title/Abstract] OR

brain ischemia*[Title/Abstract] OR

brain ischaemia*[Title/Abstract] OR

brain infarction*[Title/Abstract] OR

intracranial hemorrhage*[Title/Abstract] OR

intracranial haemorrhage*[Title/Abstract] OR

intracerebral hemorrhage*[Title/Abstract] OR

intracerebral haemorrhage*[Title/Abstract] OR

intracranial embolism*[Title/Abstract] OR

intracranial thromb*[Title/Abstract] OR

lacunar infarct*[Title/Abstract] OR

lacunar stroke*[Title/Abstract] OR

poststroke[Title/Abstract] OR

post stroke[Title/Abstract] OR

brain vascular accident*[Title/Abstract] OR

cerebral infarct*[Title/Abstract] OR

brain hemorrhage*[Title/Abstract] OR

brain haemorrhage*[Title/Abstract]))

AND

("Upper Extremity"[Mesh] OR

upper extremit*[Title/Abstract] OR

Membrum superius [Title/Abstract] OR

Upper Limb*[Title/Abstract] OR

arm[Title/Abstract] OR arms[Title/Abstract] OR

hand[Title/Abstract] OR hands[Title/Abstract] OR

"Paresis"[Mesh] OR paresis[Title/Abstract] OR

pareses[Title/Abstract])

Cochrane Library

stroke or poststroke or cerebrovascular accident or cerebral ischemia or cerebral ischaemia or brain ischemia or brain ischaemia or brain infarction or intracranial hemorrhage or intracranial haemorrhage or intracerebral hemorrhage or intracerebral haemorrhage or intracranial embolism or intracranial thromb* or brain vascular accident or cerebral infarct or brain hemorrhage or brain haemorrhage

Repetitive transcranial magnetic stimulat* or TMS or rTMS or TBS or iTBS or theta burst stimulation or θ burst stimulation

Upper Extremity or upper extremit* or arm or arms or hand or hands or Paresis or paresis or pareses or upper limb*

Embase

'cerebrovascular accident'/exp OR 'brain ischemia'/exp OR 'brain hemorrhage'/exp OR 'cerebrovascular accident': ti, ab OR 'cerebral ischemia': ti, ab OR 'cerebral ischaemia': ti, ab OR 'brain ischemia': ti, ab OR 'brain ischaemia': ti, ab OR 'brain infarction': ti, ab OR 'intracranial hemorrhage': ti, ab OR 'intracranial haemorrhage': ti, ab OR 'intracerebral hemorrhage': ti, ab OR 'intracerebral haemorrhage': ti, ab OR 'intracranial embolism':ti,ab OR 'intracranial thrombus': ti, ab OR 'stroke': ti, ab OR 'poststroke': ti, ab OR 'brain vascular accident': ti, ab OR 'cerebral infarct': ti, ab OR 'brain hemorrhage': ti, ab OR 'brain haemorrhage': ti, ab

AND

'transcranial magnetic stimulation'/exp OR 'Repetitive transcranial magnetic stimulat*': ti, ab OR ' TMS': ti, ab OR 'rtms': ti, ab OR 'tbs': ti, ab OR 'itbs': ti, ab OR 'theta burst': ti, ab OR 'θ burst': ti, ab

AND

'upper limb'/exp OR 'upper limb function': ti, ab OR 'upper extremity': ti, ab OR 'upper extremities': ti, ab OR 'arm': ti, ab OR 'arms': ti, ab OR 'hand': ti, ab OR 'hands': ti, ab OR 'paresis': ti, ab OR 'pareses': ti, ab

Web of Science

#1 TS =(Stroke OR Brain Ischemia OR Intracranial Hemorrhages OR CVA* OR cerebrovascular accident* OR cerebral ischemia* OR cerebral ischaemia* OR brain ischemia* OR brain ischaemia* OR brain infarction* OR intracranial hemorrhage* OR intracranial haemorrhage* OR intracerebral hemorrhage* OR intracerebral haemorrhage OR intracranial embolism OR intracranial thromb* OR poststroke OR brain vascular accident* OR cerebral infarct* OR brain hemorrhage* OR brain haemorrhage*)

#2 TS =(Repetitive Transcranial Magnetic Stimulat* OR TMS OR rTMS OR TBS OR iTBS OR theta burst stimulation OR θ burst stimulation)

#3 TS =(Upper Extremity OR upper extremit* OR Membrum superius OR Upper Limb* OR arm OR arms OR hand OR hands OR paresis OR pareses)

#1 and #2 and #3
